# Bias in effect size of systemic lupus erythematosus susceptibility loci across Europe: a case-control study

**DOI:** 10.1186/ar3818

**Published:** 2012-04-27

**Authors:** Elisa Alonso-Perez, Marian Suarez-Gestal, Manuel Calaza, Gian Domenico Sebastiani, Rudolf Pullmann, Chryssa Papasteriades, Attila Kovacs, Fotini N Skopouli, Marc Bijl, Ana Suarez, Maurizio Marchini, Sergio Migliaresi, Patricia Carreira, Josep Ordi-Ros, Torsten Witte, Sarka Ruzickova, Maria Jose Santos, Nadia Barizzone, Francisco J Blanco, Bernard R Lauwerys, Juan J Gomez-Reino, Antonio Gonzalez

**Affiliations:** 1 Laboratorio de Investigacion 10 and Rheumatology Unit, Instituto de Investigacion Sanitaria - Hospital Clinico Universitario de Santiago, Choupana s/n, Santiago de Compostela 15706, Spain; 2 U.O. Complessa di Reumatologia, Azienda Ospedaliera San Camillo - Forlanini, Piazza Carlo Forlanini 1, Rome 00151, Italy; 3 Institute of Clinical Biochemistry, Martin Faculty Hospital and Jessenius Medical Faculty, Kollárova 2, Martin 036 59, Slovakia; 4 Department of Histocompatibility and Immunology, Evangelismos Hospital, Ipsilantou Str. 45-47, Athens 10675, Greece; 5 Department of Rheumatology, Hospital of Hungarian Railways, Lukács György utca 4, Szolnok 5000, Hungary; 6 Pathophysiology Department, Athens University Medical School, Korai 5, Athens 115 27, Greece; 7 Department of Internal Medicine and Rheumatology, Martini Hospital, Van Swietenplein 1, Groningen 9728, the Netherlands; 8 Department of Functional Biology, University of Oviedo, Calle Catedrático Valentín Andrés Álvarez s/n, Oviedo 33006, Spain; 9 Referral Center for Systemic Autoimmune Diseases, Fondazione IRCCS Ca' Granda Ospedale Maggiore Policlinico and University of Milan, Via Francesco Sforza 35, Milan 20122, Italy; 10 Rheumatology Unit, Second University of Naples, Via Sergio Pansini 5, Naples 80131, Italy; 11 Rheumatology Department, Hospital 12 de Octubre, Av. Andalucía s/n, Madrid 28041, Spain; 12 Internal Medicine and Research Laboratory in Autoimmune Diseases, Hospital Vall d'Hebron, Passeig Vall d'Hebron 119-129, Barcelona 08035, Spain; 13 Department of Internal Medicine and Division of Clinical Immunology, Hannover Medical School, Carl-Neuberg-Straße 1, Hannover 30625, Germany; 14 Institute of Biotechnology, Academy of Sciences of the Czech Republic, Národní 1009/3, Prague 110 00, Czech Republic; 15 Rheumatology Department, Hospital Garcia de Orta and Rheumatology Reseach Unit, Instituto de Medicina Molecular, Av. Torrado da Silva s/n, Lisboa 2801-951, Portugal; 16 Department of Medical Sciences and IRCAD, Eastern Piedmont University, Via dei Tornielli 12, Novara 28100, Italy; 17 Laboratorio de Investigación Osteoarticular y del Envejecimiento, Servicio de Reumatología, INIBIC-CH Universitario, Jubias de Arriba 84A, A Coruña 15006, Spain; 18 Cliniques Universitaires Saint-Luc, Université catholique de Louvain, Avenue Emmanuel Mounier 81, Brussels 1200, Belgium; 19 Department of Medicine, University of Santiago de Compostela, Calle Choupana s/n, Santiago de Compostela 15706, Spain

## Abstract

**Introduction:**

We aimed to investigate whether the effect size of the systemic lupus erythematosus (SLE) risk alleles varies across European subpopulations.

**Methods:**

European SLE patients (*n *= 1,742) and ethnically matched healthy controls (*n *= 2,101) were recruited at 17 centres from 10 different countries. Only individuals with self-reported ancestry from the country of origin were included. In addition, participants were genotyped for top ancestry informative markers and for 25 SLE associated SNPs. The results were used to compare effect sizes between the Central Eureopan and Southern European subgroups.

**Results:**

Twenty of the 25 SNPs showed independent association with SLE, These SNPs showed a significant bias to larger effect sizes in the Southern subgroup, with 15/20 showing this trend (*P *= 0.019) and a larger mean odds ratio of the 20 SNPs (1.46 vs. 1.34, *P *= 0.02) as well as a larger difference in the number of risk alleles (2.06 vs. 1.63, *P *= 0.027) between SLE patients and controls than for Central Europeans. This bias was reflected in a very significant difference in the cumulative genetic risk score (4.31 vs. 3.48, *P *= 1.8 × 10^-32^). Effect size bias was accompanied by a lower number of SLE risk alleles in the Southern subjects, both patients and controls, the difference being more marked between the controls (*P *= 1.1 × 10^-8^) than between the Southern and Central European patients (*P *= 0.016). Seven of these SNPs showed significant allele frequency clines.

**Conclusion:**

Our findings showed a bias to larger effect sizes of SLE loci in the Southern Europeans relative to the Central Europeans together with clines of SLE risk allele frequencies. These results indicate the need to study risk allele clines and the implications of the polygenic model of inheritance in SLE.

## Introduction

The systemic lupus erythematosus (SLE) genetic component has been partially elucidated thanks to large studies that have uncovered more than 30 loci reaching very convincing disease association [1-12]. These studies have shown that a large fraction of the SLE loci (such as *STAT4*, *TNFSF4 *or *BLK*) are shared in the different ethnic groups; however, other loci are not (such as *PTPN22*, which is exclusive of Europeans). These latter loci can be due to the absence or rarity of the polymorphism in one of the ethnic groups (as for *PTPN22*, which is absent in Asians), but other SLE loci show a similar frequency in discordant populations (as for *PXK *or *FCGR2A*). Possible explanations for these conflicting results have been envisaged, including differences in linkage between the causal polymorphism and the analysed SNPs, limitations of study design, or differences in the interactions with other genetic loci or with environmental exposures [13].

Gene-gene interaction could be behind the observation that SLE loci show variability in effect sizes in function of the genetic background. For example, the Amerindian genetic background is associated with a higher effect size of several SLE loci in Hispanics [14]. We wondered whether genetic heterogeneity of this type could exist among European subjects. Support for this hypothesis is provided by the recent evidence of differences in SLE clinical features among Europeans [15-17] and by opposed results of SLE association with *PDCD1 *[18,19]. Notably, these two observations showed a North-South axis of variations, which is the main axis of European population differentiation [20-23].

Our study included 25 top-associated SNPs in the better known SLE loci studied in 1,742 patients with SLE and in 2,101 controls from 17 collections recruited in 10 European countries, each of them with homogeneous local ancestry. The results showed a bias to larger effect sizes of the risk alleles in the Southern Europeans relative to the Central Europeans. We also found clines of risk allele frequencies.

## Materials and methods

### Patient data

We used DNA samples from European SLE patients and ethnically matched healthy controls recruited at 17 centres from 10 different countries (Table 1). Most of these samples have already been described [24,25]. Each recruiting centre was asked for about 100 patients with SLE according to the revised American College of Rheumatology classification criteria [26] and for about 100 controls, producing a total of 1,742 cases and 2,101 controls. Only individuals with self-reported ancestry from the country of origin were included. All participants gave their written informed consent to participate and the study was approved by the relevant ethics committees.

**Table 1:** Sample collections, proportion of women, frequencies of the three more informative AIMs and North/South score

	DNA sample		Women (%)		AIM frequency (%)			N/S score
		
Collection^a^	Control	SLE	Control	SLE	*rs12913832*	*rs382259*	*rs6730157*	
NL	180	104	59.4	86.5	83.0	77.6	72.5	100.0
BE^b^	106	147	93.5	90.5				
DE	95	90	n.a.	90	77.9	68.1	56.9	80.5
CZ^b^	100	101	32	85.1				
SK	93	94	93.5	91.5	72.8	63.8	39.4	64.4
HU	95	95	48.4	90.4	63.4	59.6	39.0	54.9
ES, LCG	145	88	82.9	92	35.4	67.4	45.3	47.6
ES, OVD	200	147	69	91.8	30.5	70.4	45.4	47.2
ES, SCQ	95	109	48.4	96	35.1	68.2	41.3	46.0
ES, BCN	97	90	52.6	91.1	32.6	63.2	41.6	40.5
ES, MAD	281	92	68.8	91.3	33.1	61.9	42.3	40.0
PT	97	100	91.3	95.3	28.7	60.7	39.1	34.6
IT, MXP	106	129	42.5	86.8	47.6	49.1	19.8	26.2
IT, ROM	102	84	55.9	89.7	38.2	51.9	15.3	20.3
GR, AUMS	100	95	92	91.6	36.0	44.0	13.4	11.3
IT, NAP	109	79	100	90.1	38.6	42.2	10.0	9.6
GR, EH	100	98	67	86.7	36.7	38.0	13.5	6.8

Collections are in descending order of N/S score. ^a^Collections from: University Medical Center Groningen, The Netherlands (NL); Université Catholique de Louvain, Belgium (BE); Hannover Medical School, Germany (DE); Institute of Rheumatology, The Czech Republic (CZ); Martin Faculty Hospital, Slovakia (SK); Albert Szent-Györgyi Medical and Pharmaceutical Centre, Hungary (HU); Complexo Hospitalario Universitario A Coruña, Spain (ES, LCG); Hospital Universitario Central de Asturias, Spain (ES, OVD); Hospital Clinico Universitario de Santiago, Spain (ES, SCQ); Hospital Val d'Hebron of Barcelona, Spain (ES, BCN); Hospital 12 de Octubre, Madrid, Spain (ES, MAD); Hospital Garcia de Orta, Almada, Portugal (PT); University of Milan and Fondazione IRCCS Ospedale Maggiore Policlinico from Milan, Italy (IT, MXP), Ospedale S. Camillo - Forlanini from Roma, Italy (IT, ROM); Athens University Medical School, Athens, Greece (GR, AUMS); Second University of Naples, Italy (IT, NAP); and Evangelismos Hospital, Athens, Greece (GR, EH). ^b^These two collections were not analysed further because of differences in AIM frequencies between cases and controls.

### Genotyping

DNA samples were amplified in a multiplex PCR with the KAPA2G fast HotStart (Kapa Biosystems, Woburn, MA, USA) in a final volume of 10 μl (20 ng genomic DNA), using 3 mM MgCl_2 _and 0.2 μM each primer. Products were purified by Exo-SAP digestion with exonuclease I (Epicentre, Madison, WI, USA) and shrimp alkaline phosphatase (GE Healthcare, Barcelona, Spain). Subsequently, single-base extension reactions were performed with the SNaPshot Multiplex kit (Applied Biosystems, Foster City, CA, USA). The genotyping call rate success of the newly studied SNPs was 99.12%. Sequences of primers and probes are available from the authors upon request.

### Selection of SNPs

Six ancestry informative markers (AIMs) were selected (see Table S1 in Additional file 1). Three of these are the most informative AIMs in differentiating Northern Europeans from Southern Europeans according to a large study [22]. Another two AIMs are the most informative for East-West place of origin according to the same study [22]. *rs12913832 *is a SNP associated with large differences in frequency across Europe and unrelated to the previous [23]. In addition, we used genotype data from another 25 SNPs tagging 22 SLE loci reported in large European studies (see Table S1 in Additional file 1). These included nine SNPs in nine SLE loci we had already replicated and that made up the first phase of the current study [24]. We selected 14 additional SNPs for *de novo *genotyping in our samples. These SNPs were the top SLE-associated SNPs in large previous studies [2,3,7,27,28]. Not all of them have reached a genome-wide association level, but they are considered solid because they were found in large studies and with an odds ratio (OR) of the risk allele > 1.15 in at least one study. These 14 SNPs together with two *IRF5 *SNPs we had already studied [25] made up the 16 SNPs included in the second phase of our study.

### Statistical analysis

Analysis of results was based on R and Statistica 7.0 (StatSoft, Tulsa, OK, USA). Conformity with Hardy-Weinberg equilibrium was tested in control samples. Allele frequencies of the AIMs were compared between patients and controls from each collection with 2 × 2 contingency tables. We created a global score by sample collection for the North-South axis of European population differentiation (N/S score) with the allele frequencies of the AIMs. First, we defined AIM allele frequencies as a function of the allele more common in Northern European populations. The most informative, nonredundant three AIMs were then selected. Finally, as a normalisation step we rescaled the frequencies from each of these three AIMs to 0 to 100%. This was done by considering 0% the frequency in the sample collection where it was less abundant and 100% the frequency where it was most abundant. The rescaled values of the three AIMs were averaged to obtain a combined normalised unique score for each collection.

Case-control allele frequencies were compared with fixed-effects and random-effects models stratifying by sample collection. For the fixed-effect model, the Mantel-Haenszel approach was used. For the random-effect model, an inverse variance meta-analysis approach was followed. Heterogeneity of effect sizes was evaluated with the inconsistency parameter *I*^2 ^derived from the Cochran Q statistic. A high, moderate and low level of inconsistency was attributed to levels of *I*^2 ^over 75%, 50% and 25%, respectively, as described previously [29].

Distributions of ORs in Central European and Southern European populations were compared with the binomial distribution. Geometric mean (*G*_mean_) values of the ORs were obtained and compared. The sum of SLE risk alleles was obtained for the 20 SNPs showing independent association with SLE. The sum of genetic risk scores (GRSs) was also calculated for the same 20 SNPs. The total GRS for each patient with SLE was the sum of the products of the natural logarithm of the OR by the number of risk alleles at each locus that was carried by this patient. The ORs used to calculate GRS were the specific Mantel-Haenszel ORs for the corresponding European subgroup. The G_mean _OR, mean of sum of risk alleles and mean cumulative GRS values were compared between groups with Student *t *tests. Correlation between the N/S score and sum of risk alleles or the mean of the natural logarithm of the OR was analysed with the weighted Pearson correlation coefficient. The threshold for significance was set at *P *≤ 0.05.

## Results

### Analysis of population differentiation

Our study included samples from 1,742 patients with SLE and 2,101 healthy controls recruited in 17 centres (Table 1). Recruiters at each centre asked the patients and the controls for their ancestry, and only those reporting uniform known ancestry from the respective country were included. In addition, we checked with six top AIMs informative for European population differentiation whether there were differences between cases and controls from each recruitment centre. Five of the six AIMs provided completely independent information (pairwise *r*^2 ^between them < 0.03) - only *rs6730157 *and *rs4988235 *were redundant (*r*^2 ^= 0.85). This analysis showed significant differences in the samples from two collections, the Czech Republic and Belgium. Consequently, these two collections of samples were discarded from all subsequent analyses.

Next, we divided the remaining 15 collections following the major axis of European population differentiation, the North-South axis. According to previous studies we expected two groups [20-22]: one with all samples from Central Europe, with samples from the Netherlands, Germany, Hungary and Slovakia (383 patients with SLE and 463 healthy controls); and a second with all samples from Portugal, Spain, Italy and Greece (1,111 patients and 1,432 controls). The AIM frequencies in samples from each collection were congruent with this division (Table 1). We obtained the N/S score (for the place of each collection on the North-South axis), which was as expected from geographical distribution and previous studies [20-22] and was in agreement with the division we made (Table 1).

### Nine SLE susceptibility loci with a Southern bias

**Figure 1:**
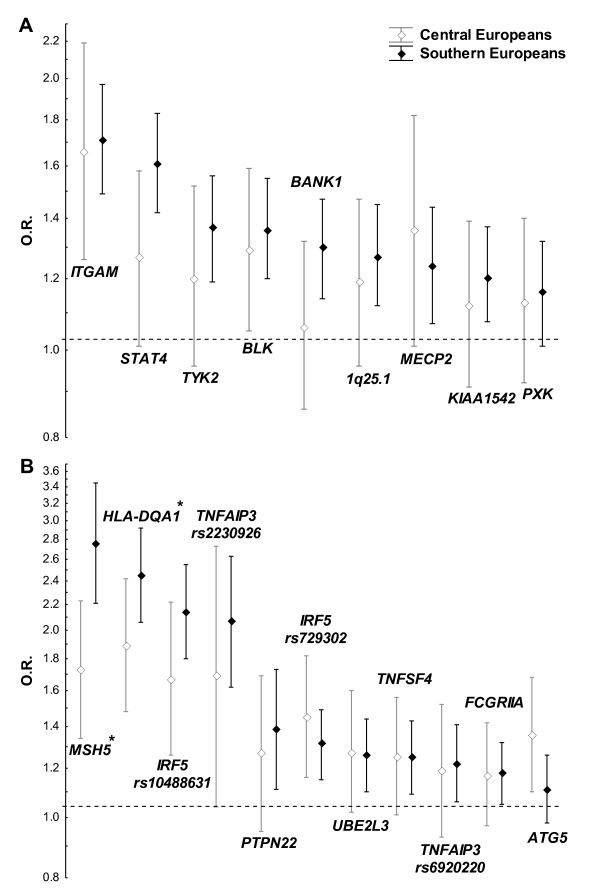
**Bias for a stronger association in Southern Europeans than in Central Europeans**. **(A) **Nine systemic lupus erythematosus (SLE) loci from Suarez-Gestal and colleagues [24]. **(B) **Eleven newly studied SLE-associated SNPs. Mantel-Haenszel odds ratio (OR) for the risk allele and their 95% confidence interval presented in descending order from left to right. *Loci showed significant differences between the two groups. *MECP2 *data only from women because it maps to the X chromosome. SNPs used for each locus are detailed in Table 2.

We have already replicated association of top SNPs in nine SLE susceptibility loci in the samples included in this study (Table 2) [24]. When these SNPs were analysed separately in the two subgroups, Central European and Southern European, we found a bias for stronger association in the latter (Figure 1A). This bias was observed in eight of the nine SNPs. This distribution is significantly different from that expected by chance (*P *= 0.039), and was observed with the Mantel-Haenszel OR (Figure 1A) and with the random-effect meta-analysis OR (see Figure S1 in Additional file 1). Mantel-Haenszel analysis was preferred because none of the 18 analyses showed high heterogeneity and only three showed a moderate level of inconsistency. It should be noted that none of the SNPs taken individually was significantly different between the two groups.

**Table 2:** Association results of the 25 systemic lupus erythematosus SNPs

	Mantel-Haenszel analysis		
		
SNP (locus)	OR (95% CI)	*P *value	Reference
*rs1143679 *(*ITGAM*)	1.70 (1.50 to 1.93)	5.16 × 10^-17^	[24]
*rs7574865 *(*STAT4*)	1.52 (1.36 to 1.70)	6.11 × 10^-14^	[24]
*rs13277113 *(*C8orf13-BLK*)	1.34 (1.20 to 1.50)	1.33 × 10^-7^	[24]
*rs2304256 *(*TYK2*)	1.32 (1.18 to 1.47)	1.17 × 10^-6^	[24]
*rs17435 *(*MECP2*)^a^	1.27 (1.11 to 1.45)	5.57 × 10^-4^	[24]
*rs10798269 *(*1q25.1*)	1.25 (1.11 to 1.39)	6.12 × 10^-5^	[24]
*rs17266594 *(*BANK1*)	1.23 (1.10 to 1.37)	1.92 × 10^-4^	[24]
*rs4963128 *(*KIAA1542*)	1.19 (1.08 to 1.32)	1.31 × 10^-3^	[24]
*rs6445975 *(*PXK*)	1.15 (1.03 to 1.27)	0.02	[24]
*rs3131379 *(*MSH5*)	2.25 (1.89 to 2.68)	6.41 × 10^-20^	-
*rs2187668 *(*HLA-DQA1*)	2.17 (1.88 to 2.51)	1.09 × 10^-25^	-
*rs10488631 *(*IRF5*)	2.00 (1.73 to 2.32)	8.38 × 10^-21^	[25]
*rs2230926 *(*TNFAIP3*)	1.99 (1.60 to 2.47)	2.54 × 10^-10^	-
*rs729302 *(*IRF5*)	1.35 (1.20 to 1.49)	1.70 × 10^-7^	[25]
*rs2476601 *(*PTPN22*)	1.34 (1.13 to 1.60)	9.15 × 10^-4^	-
*rs5754217 *(*UBE2L3*)	1.26 (1.13 to 1.42)	7.30 × 10^-5^	-
*rs2205960 *(*TNFSF4*)	1.25 (1.11 to 1.40)	1.32 × 10^-4^	-
*rs6920220 *(*TNFAIP3*)	1.21 (1.07 to 1.37)	2.25 × 10^-3^	-
*rs844644 *(*TNFSF4*)	1.19 (1.08 to 1.32)	7.22 × 10^-4^	-
*rs1801274 *(*FCGR2A*)	1.18 (1.07 to 1.30)	1.27 × 10^-3^	-
*rs573775 *(*ATG5*)	1.17 (1.05 to 1.31)	4.35 × 10^-3^	-
*rs10156091 *(*ICA1*)	1.09 (0.93 to 1.28)	0.3	-
*rs4240671 *(*XKR6*)	1.09 (0.98 to 1.19)	0.1	-
*rs2667978 *(*LYN*)	1.06 (0.94 to 1.19)	0.3	-
*rs6922466 *(*PERP*)	1.02 (0.91 to 1.14)	0.8	-

The first nine SNPs were analysed in the first phase of the study and the remaining 16 in the second. Results of Mantel-Haenszel analysis are shown. All odds ratios (ORs) are expressed for the risk allele. Analyses of some of the SNPs in these samples have already been reported [24,25]. CI, confidence interval. ^a^Analysis performed only in women because the locus is in the X chromosome.

### Association analysis of additional SLE loci

We wanted to assess whether the bias found was a general phenomenon of SLE loci. We therefore selected 16 SNPs identifying other SLE genetic loci in Europeans [2,3,7,27,28]. All of the SNPs were genotyped successfully and all were in Hardy-Weinberg equilibrium when analysed by collection (the two *IRF5 *SNPs have already been studied in a fraction of the samples [25]). The combined data showed significant differences between SLE cases and controls for 12 SNPs (Table 2). All of the significant differences were in the same direction as originally reported. Only four SNPs were similar in cases and controls, so they were excluded from further analysis. To avoid redundancy, we checked with conditional logistic regression whether the two associated SNPs in *IRF5*, *TNFAIP3*, *TNFSF4 *or the two SNPs in the HLA (*rs2187668 *and *rs3131379*) contributed independently to the association. One of the *TNFSF4 *SNPs (*rs844644*) showed no association when conditioned in the other *TNFSF4 *SNP (*P *= 0.072), and therefore was no longer considered. On the contrary, the two *IRF5*, the two *TNFAIP3 *and the two HLA SNPs remained associated and were included in the following analyses.

Stratification of the SLE patients and controls in Central Europeans and Southern Europeans showed that two of the 11 SNPs (*rs3131379 *in *MSH5*, *P *= 0.003; and *rs2187668 *in *HLA-DQA1*, *P *= 0.046) were significantly more associated in the Southern subgroup than in the Central European subgroup in the Mantel-Haenszel meta-analysis (Figure 1B); but none was significantly different in the random-effects meta-analysis (see Figure S2 in Additional file 1). The Mantel-Haenszel meta-analysis was favoured because none of the 22 analyses showed high inconsistency and only five showed a moderate level. In total, seven of the 11 associated SNPs were numerically more associated in the Southern European subgroup (Figure 1B). Only three SNPs were more associated in the Central Europeans (*rs573775 *in *ATG5*, *rs729302 *in *IRF5*, and *rs5754217 *in *UBE2L3*) and one was equally associated in the two subgroups (*rs2205960 *in *TNFSF4*), but none of these differences were significant.

### Southern bias for all of the SLE-associated SNPs together

When the 20 SNPs (nine from the first phase and 11 from the second) that have shown independent association in our samples were considered together, a bias towards a stronger association in the Southern subgroup was observed both as a significant deviation of the OR from a random binomial distribution (*P *= 0.019) and as a significant difference between the OR means, which was larger in the Southern samples (*G*_mean _= 1.46 ± 1.30) than in the Central European samples (*G*_mean _= 1.34 ± 1.17, *P *= 0.02). Because it was possible that a fraction of the effect size attributed to a SNP is dependent on other SLE-associated SNPs, we also compared the mean of the OR for each SNP conditional on all the other SNPs. This analysis also showed a larger effect size in the Southern subjects (*G*_mean _= 1.43 ± 1.24) than in the Central Europeans (*G*_mean _= 1.28 ± 1.18, *P *= 0.02).

**Figure 2:**
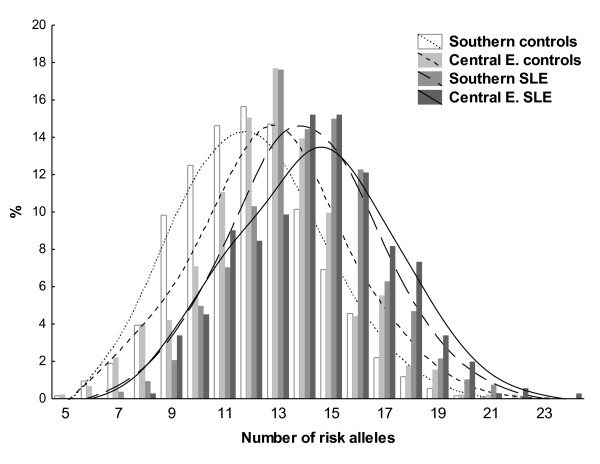
**Systemic lupus erythematosus risk alleles in Central and Southern European patients and controls**. Distribution of the number of systemic lupus erythematosus (SLE) risk alleles in Central European and Southern European SLE patients and controls. *y *axis, percentage of subjects in each of the four strata with the corresponding total number of SLE risk alleles. Distance-weighted least-squares fitting lines are presented. White bars and dotted line, Southern European controls; light grey bars and short-slashed line, Central European controls; medium grey bars and long-slashed line, Southern European SLE patients; dark grey bars and continuous line, Central European SLE patients.

We also analysed our data in a different way by counting the SLE risk alleles carried by each subject. Although it was possible to have from zero to 40 risk alleles, none of the subjects had less than five or more than 24 risk alleles. The distribution of frequencies stratified by disease status and by Central versus Southern subpopulations showed a gradient of values (Figure 2). The lowest number of risk alleles was observed in the healthy controls from the Southern European group (mean ± standard deviation = 12.0 ± 2.5). Immediately higher was the number of risk alleles corresponding to the Central European controls (12.8 ± 2.7, *P *= 1.1 × 10^-8 ^vs. the Southern European controls). This group was followed for the Southern European SLE patients (14.0 ± 2.5, *P *= 2.0 × 10^-17 ^vs. the Central European controls) and, finally, for the Central European patients (14.4 ± 2.8, *P *= 0.016 vs. the Southern SLE patients).

The differences in number of risk alleles were not due to confounding by deviations from Hardy-Weinberg equilibrium in any of the four sample groups for any of the SNPs or by differences in call rate between the four groups (data not shown). We also checked that the differences and order hold when only women were analysed, with the lowest number in Southern controls (12.0 ± 2.4) followed by Central controls (13.0 ± 2.6), Southern SLE patients (14.1 ± 2.5) and Central European patients (14.4 ± 2.8) - showing significant differences between each of these groups except the last two (*P *= 5.3 × 10^-9^, *P *= 2.1 × 10^-8 ^and *P *= 0.056, respectively). The same sequence was observed when the comparison was made in men (Southern controls 12.0 ± 2.7 and Central controls 12.7 ± 3.0, *P *= 0.01; Southern SLE patients 13.5 ± 2.5, *P *= 0.036; Central SLE patients 14.7 ± 2.8, *P *= 0.027). In this case, the differences between groups were all significant in spite of the small size of the SLE patient groups.

These results showed from a different perspective the same bias in effect sizes that has been described in the previous paragraphs, because the difference in number of risk alleles between SLE patients and controls of Southern origin (difference = 2.06, 95% confidence interval = 1.85 to 2.26) was significantly larger than for patients and controls of Central European origin (difference = 1.63, 95% confidence interval = 1.25 to 2.01; *P *= 0.027). In addition, these results showed that the SLE risk alleles were less frequent in the Southern European subjects overall, but the difference was significantly more marked between controls from the Southern and Central subgroups (difference = 0.81, 95% confidence interval = 0.53 to 1.09) than between SLE patients from the same subgroups (difference = 0.38, 95% confidence interval = 0.07 to 0.69; *P *= 0.023).

**Figure 3:**
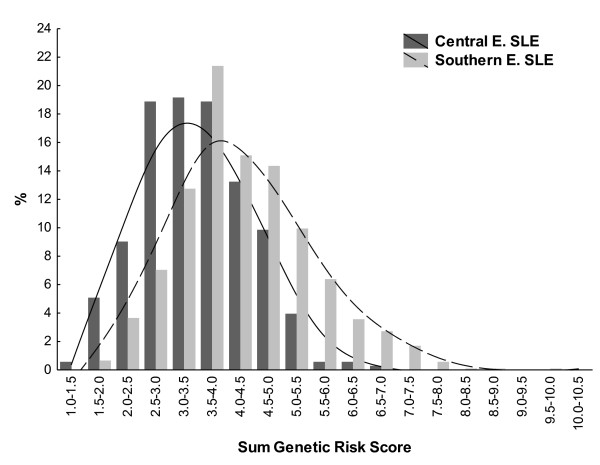
**Sum of genotypic risk score in Central and Southern European patients with systemic lupus erythematosus**. *y *axis, percentage of subjects in each of the two groups with sum genetic risk score (GRS) over 20 SNPs in the indicated intervals. Distance-weighted least-squares fitting lines are presented. Light grey bars and long-slashed line, Southern European systemic lupus erythematosus (SLE) patients; dark grey bars and continuous line, Central European SLE patients.

We also compared the cumulative GRS between Southern and Central European SLE patients. This parameter includes information from the sum of risk alleles and from the OR, and therefore is not independent of previous comparisons (Figure 3). The mean sum GRS was significantly larger in Southern European patients than in Central European patients (4.31 ± 1.17 vs. 3.48 ± 0.93, *P *= 1.8 × 10^-32^). The difference persisted after excluding the two HLA SLE-associated SNPs from the analysis (3.74 ± 0.84 vs. 2.92 ± 0.68, *P *= 2.4 × 10^-58^).

**Figure 4:**
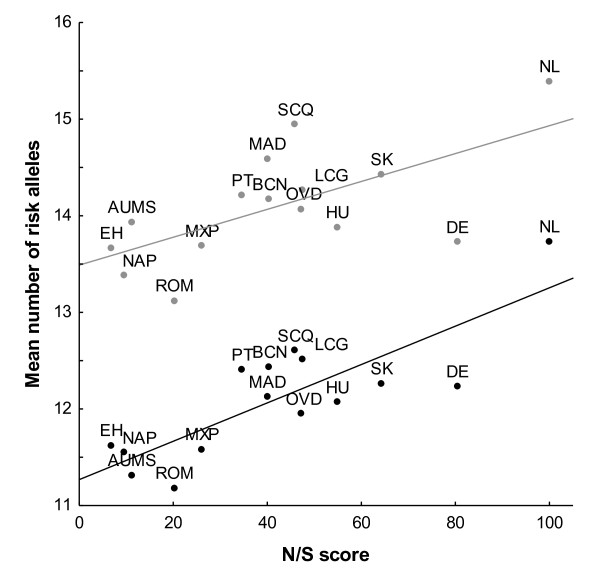
**Correlation between North-South population differentiation axis and number of systemic lupus erythematosus risk alleles**. Mean number of SLE risk alleles per sample collection is represented against the North/South score (N/S score) obtained in the same samples. Collection letter codes are as in Table 1.

Finally, we wanted to analyse the mean number of SLE risk alleles for each sample collection as a function of its position along the North-South axis of population differentiation (Figure 4). This analysis showed that the number of SLE risk alleles in controls and in patients with SLE increased with the N/S score (*R*^2 ^= 0.67, *P *= 0.002 for controls; and *R*^2 ^= 0.45, *P *= 0.002 for patients). Similarly, the mean of the natural logarithm of OR correlated with the N/S score (*R*^2 ^= 0.40, *P *= 0.012). The gradual change in function of the score indicates that the previously performed analyses were not sensitive to the point used to separate Central European from Southern European populations.

### Frequency clines of the SLE risk alleles

The previous results suggest the possibility of frequency gradients or clines of SLE risk alleles along the North-South axis of population differentiation. We therefore analysed this possibility for each of the SNPs. For this analysis we only used data from controls because they are more representative of the general population. None of the SLE-associated SNPs showed significant linkage disequilibrium with the AIMs included in this study (all pairwise *r*^2 ^between AIMs and SLE-associated SNPs < 0.05). However, 10 SNPs were significantly different between Central Europeans and Southern Europeans (Table 3). Five of these showed differences in excess of 5%, including the two SNPs in the *HLA *region, *PTPN22 *(which is already known to show a cline in Europe [30]), *BLK *and *PXK*. Eight of the 10 SNPs were more common in controls from the Central group than from the Southern group in accordance with the direction of change observed with the sum of all risk alleles. The two exceptions were the *ITGAM *and *FCGR2A *SNPs. These two SNPs, however, have shown the same bias in effect sizes as the other eight. We also checked how the SNP frequencies in controls fitted a linear regression as a function of the N/S score. Results were similar to the obtained with the Central versus Southern group comparisons, except for SNPs with modest differences and for the SNP in *BLK *(Table 3).

**Table 3:** Risk allele frequencies in Central and Southern European controls and relationship with the North/South score

	RAF (%)			RAF vs. N/S score		
	
SNP (locus)	Central European	Southern European	χ^2 ^*P *value	*r_xy_*	Slope	*P *value
*rs3131379 *(*MSH5*)	14.9	4.6	4.9 × 10^-26^	0.92	4.15	2.0 × 10^-6^
*rs2187668 *(*HLA-DQA1*)	15.7	8.7	1.9 × 10^-9^	0.88	4.93	4.0 × 10^-5^
*rs2476601 *(*PTPN22*)	11.8	6.1	1.5 × 10^-8^	0.74	4.21	2.7 × 10^-3^
*rs13277113 *(*C8orf13-BLK*)	31.0	23.2	2.2 × 10^-6^	0.20	0.95	NS
*rs6445975 *(*PXK*)	29.0	22.6	7.5 × 10^-5^	0.66	2.67	0.01
*rs1143679 *(*ITGAM*)	11.9	16.7	4.9 × 10^-4^	-0.59	-4.79	0.03
*rs2205960 *(*TNFSF4*)	25.2	20.7	4.9 × 10^-3^	0.15	1.08	NS
*rs1801274 *(*FCGR2A*)	44.5	48.6	0.03	-0.40	-1.25	NS
*rs4963128 *(*KIAA1542*)	69.1	65.3	0.03	0.58	2.83	0.03
*rs10488631 *(*IRF5*)	11.6	9.3	0.046	0.46	3.64	0.10
*rs17435 *(*MECP2*)^a^	20.9	22.8	NS	-0.57	-2.84	0.03
*rs573775 *(*ATG5*)	28.2	26.3	NS	0.51	4.10	0.06
*rs7574865 *(*STAT4*)	23.3	23.8	NS	-0.51	-4.63	0.06
*rs6920220 *(*TNFAIP3*)	19.3	17.8	NS	0.49	2.74	0.07
*rs10798269 *(*1q25.1*)	66.2	69.0	NS	-0.17	-1.16	NS
*rs5754217 *(*UBE2L3*)	22.8	20.8	NS	0.16	1.28	NS
*rs2304256 *(*TYK2*)	73.1	71.0	NS	0.06	0.23	NS
*rs2230926 *(*TNFAIP3*)	3.5	4.3	NS	-0.21	-3.42	NS
*rs729302 *(*IRF5*)	67.8	69.0	NS	-0.00	-0.03	NS
*rs17266594 *(*BANK1*)	71.2	70.9	NS	0.32	1.99	NS

Comparison of the risk allele frequencies (RAFs) between Central and Southern European controls, and linear regression of RAFs to the North/South score (N/S score) of population differentiation (from Table 1). NS, not significant. ^a^Analysis performed only in women because the locus is in the X chromosome.

## Discussion

Two main aspects of our results should be highlighted: a significant bias to larger effect sizes of the SLE susceptibility loci in subjects from Southern Europe than those from Central Europe, and a lower frequency of the SLE risk alleles at these loci in subjects from Southern Europe.

The bias to stronger association among Southern Europeans was shown with four types of analysis. The first, comparing the number of SLE loci showing a trend to stronger associations beyond the expected at random, is independent of the loci characteristics. In contrast, comparison of the mean OR reflects the magnitude of the differences in effect size. An advantage of these two analyses is that they were done with meta-analysis approaches to account for sample collection factors. Their limitation is that they convey little information about the nature of the differences. The next analysis, comparison of the number of SLE risk alleles carried by each subject, is more informative but does not account for sample collection effects. The last analysis, comparison of sum GRS, combines information from the two previous analyses and is therefore not independent. Concordance of results from the different analyses is reassuring.

Further confidence was gained from the gradual change of the number of SLE risk alleles per sample collection as a function of the N/S score, implying insensitivity of the results to the specific partition of Europeans we have used. It is also important to note that the studies which identified SLE risk loci were carried out with subjects of a dominant Northern European ancestry (full references in Table S1 in Additional file 1), and therefore the stronger association we have found in Southern Europeans cannot be attributed to ascertainment bias. In other words, any bias due to the discovery of SLE loci in Northern-Central Europeans will favour a stronger effect size in that subpopulation, which is the opposite of our findings. This makes it very unlikely that our results are due to a tighter linkage disequilibrium between causal SNPs and the studied SNPs in the Southern subjects. All of these considerations support the validity of the our findings. One should note that it was an average effect, however, because not all of the SLE risk alleles showed a trend to stronger effect sizes in the Southern samples and, in most of those that showed the trend, differences were small and not significant when assessed individually.

The second aspect of our results is the differential distribution of SLE risk alleles, with a lower frequency in Southern Europeans than in Central Europeans. The difference was observed both in SLE patients and in controls, but was more marked in the latter. It was maximal between the subjects from Greece and Italy, on one side, and those for the Netherlands on the other. All of the remaining groups were in between. Seven of the SNPs showed a frequency cline correlating with the N/S score. Only one of these clines has already been described for the risk allele of *PTPN22 *[30].

The existence of allele frequency clines in the European population and their main axis of differentiation are well established [20-23]. What is surprising about our results is that the number of SLE risk alleles followed a gradient along this axis instead of varying randomly, with some alleles more common towards the North and others towards the South. This observation suggests the possible effect of selective forces acting along the history of the European populations, probably through resistance to infections [31,32]. These ideas demand new studies aiming to explore the relationship of autoimmunity with infection vulnerability.

A notable facet of the cline of SLE risk alleles in our study was that the difference between Southern Europeans and Central Europeans is less marked in SLE patients than in controls. We propose that this is due to the genetic structure of SLE that makes patients more similar at SLE loci across European subpopulations than the average member of the same subpopulations. This hypothesis is derived from the polygenic model of genetic inheritance [33], which includes the concept of liability threshold: disease appears when the contribution of multiple genetic factors to disease liability overcomes a threshold. Diseased subjects are therefore, on average, more similar for the genes involved in the disease than are the controls. More complex scenarios with involvement of differential environmental factors are also possible, however, and the risk allele of a particular locus could follow a different pattern because of specific factors besides the generic effect proposed here.

The validity of our results will be reinforced by replication in other sample collections. This will be particularly important in relation to the Central European subgroup - this was the smallest in our study, causing lower precision in the OR values and lower power to detect differential effects. Data from additional populations would also be interesting, in particular from the more extreme European subpopulations, because coverage of the full European spectrum would allow detecting additional clines. In addition, the AIMs we used were sufficient for group-level analyses but not for classification of individual subjects, which could have made our analyses more powerful. The studied AIMs were able to show the European population substructure along the North-South differentiation, however, as in the studies where these AIMs were selected [22,23].

Our study also provides independent replication of SLE loci. Most of these loci were already strongly established and do not require comment (full references in Table S1 in Additional file 1). In contrast, four SNPs with previous solid association were not replicated in our analysis. Probably the most solid of them is *rs10156091 *in *ICA1*. This SNP was first associated with SLE in a large genome-wide association scan (GWAS; *P *= 1.9 × 10^-7^, OR = 1.32) [3]. This result was confirmed in an even larger replication study, but with a lower effect size (OR = 1.16, *P *= 6.5 × 10^-4^) [7]. The allelic frequency of this SNP in controls (10.0%) implies that our study had 97% power to detect the originally described effect with *P *< 0.05, but only 51% power for an OR like that observed in the replication study.

Also very solid is the record of *rs2667978 *in *LYN*. This was discovered in the same GWAS (*P *= 5.1 × 10^-8^, OR = 0.81) [3] and confirmed in a large replication study with European descent samples, but more weakly (*P *= 0.016) [34]. However, this association was not confirmed in the already mentioned large replication study where a different SNP was used, *rs7829816 *[7]. Our study was powered to detect an effect as that originally described with *P *< 0.01. The *rs4240671 *SNP in *XKR6 *is also backed by strong evidence. It was identified in the same SLE GWAS as *ICA1 *and *LYN *with very strong evidence including five SNPs with *P *< 5.0 × 10^-8 ^[3]. The association was not uniformly observed across sample collections in this study, however, and no replication in any large study has been reported. The only independent replication was obtained for one of the SNPs in a 245-family study in Canadians (*rs6985109*; *P *= 0.008) [35]. Our study had enough power to detect the originally reported association (OR = 0.75) with *P *< 10^-7 ^given its elevated allele frequency (49%). Finally, the weakest previous support was for *rs6922466 *in *PERP*. This SNP was associated with SLE (*P *= 1.0 × 10^-4^) only in a large study [28].

## Conclusion

Our study has uncovered a bias for stronger effect sizes of SLE risk alleles in Southern Europeans than in Central Europeans. This bias was accompanied by a lower frequency of the risk alleles in the Southern European group. Difference in frequencies was more marked in controls than in patients with SLE. These results should be taken into account for genetic studies of SLE and for understanding the genetic structure of the disease and the possible presence of autoimmune disease risk allele clines and their causes. In addition, these results call for exploration of the assumptions and implications of the liability threshold concept - in particular, whether a constant threshold is consistent with GWAS data - as well as for exploration of environmental or other factors that could explain the effect size bias. Our findings therefore contribute to define the genetic epidemiology of SLE and suggest new lines of research for understanding the deep genetic structure of SLE.

## Abbreviations

AIM: ancestry informative marker; CI: confidence interval; *G*_mean_: geometric mean; GRS: genetic risk score; GWAS: genome-wide association scan; N/S score: North/South score; OR: odds ratio; PCR: polymerase chain reaction; SLE: systemic lupus erythematosus; SNP: single nucleotide polymorphism.

## Competing interests

The authors declare that they have no competing interests.

## Authors' contributions

EA-P and MS-G participated in the design of the study, genotyped the samples, and participated in the interpretation of the results and writing the manuscript. MC participated in the statistical analysis and in the interpretation of results. GDS, RP, CP, AK, FNS, MB, AS, MM, SM, PC, JO-R, TW, SR, MJS, NB, FJB, BRL and JJG-R participated in the acquisition of clinical data and collection of samples and in the analysis and interpretation of results. AG participated in the design of the study and in coordination of the acquisition of clinical data and collection of samples, and supervised laboratory work and interpretation and writing of the manuscript. All authors read and approved the final manuscript.

## Supplementary Material

Additional file 1 **Table S1 presenting a detailed description of all the SNPs included in the study, and Figures S1 and S2 with random effect meta-analysis results corresponding to the same data as **Figure 1**in the manuscript**. Click here for file
