# Supplementary material for: Bias in effect size of systemic lupus erythematosus susceptibility loci across Europe: a case-control study
Source: Arthritis Res Ther. 2012 Apr 27;14(2):R94. doi: 10.1186/ar3818 (PMC3446468; doi:10.1186/ar3818)

**Table S1:** SNPs included in the study with indication of their alleles, chromosome, locus, location in the locus, reference from which they were obtained and their use: phase 1 includes the 9 SLE associated SNPs from Suarez-Gestal et al. [24]; phase 2 the SNPs newly analyzed in this report; and AIM the 6 European Ancestry Informative Markers

| **SNP** | **Alleles^a^** | **Chr** | **Locus** | **Location** | **Reference** | **Use** |
| --- | --- | --- | --- | --- | --- | --- |
| *rs1143679* | G/A | 16 | *ITGAM* | Exon 3 | 1 | phase 1 |
| *rs7574865* | G/T | 2 | *STAT4* | Intron 3 | 2 - 7 | phase 1 |
| *rs13277113* | G/A | 8 | *C8orf13-BLK* | Intergenic | 3 | phase 1 |
| *rs2304256* | C/A | 19 | *TYK2* | Exon 8 | 8, 9 | phase 1 |
| *rs17435* | A/T | X | *MECP2* | Intron 2 | 10 | phase 1 |
| *rs10798269* | G/A | 1 | *1q25.1* | Intergenic | 2 | phase 1 |
| *rs17266594* | T/C | 4 | *BANK1* | Intron 1 | 11 | phase 1 |
| *rs4963128* | G/A | 11 | *KIAA1542* | Intron 4 | 2 | phase 1 |
| *rs6445975* | T/G | 3 | *PXK* | Intron 5 | 2 | phase 1 |
| *rs509749* | A/G | 1 | *LY9* | Exon 8 | 12 | phase 1 |
| *rs3131379* | G/A | 6 | *MSH5* | Itron 1 | 2 | phase 2 |
| *rs2187668* | G/A | 6 | *HLA-DQA1* | Intergenic | 3 | phase 2 |
| *rs2230926* | T/G | 6 | *TNFAIP3* | Exon 3 | 13 | phase 2 |
| *rs6920220* | G/A | 6 | *TNFAIP3* | Intergenic | 14 | phase 2 |
| *rs6922466* | A/G | 6 | *PERP* | Intergenic | 13 | phase 2 |
| *rs2476601* | G/A | 1 | *PTPN22* | Exon 8 | 2, 14 | phase 2 |
| *rs844644* | C/A | 1 | *TNFSF4* | Intergenic | 15 | phase 2 |
| *rs2205960* | G/T | 1 | *TNFSF4* | Intergenic | 14, 15 | phase 2 |
| *rs1801274* | G/A | 1 | *FCGRIIA* | Exon 4 | 2, 14 | phase 2 |
| *rs10156091* | C/T | 7 | *ICA1* | Intron 5 | 2, 14 | phase 2 |
| *rs4240671* | A/G | 8 | *XKR6* | Intron 1 | 2 | phase 2 |
| *rs2667978* | A/G | 8 | *LYN* | Intron 8 | 2 | phase 2 |
| *rs5754217* | G/T | 22 | *UBE2L3* | Intron 1 | 2, 14 | phase 2 |
| *rs573775* | G/A | 6 | *ATG5* | Intron 7 | 2 | phase 2 |
| *rs6730157* | A/G | 2 | *RAB3GAP1* | Intron 22 | 16 | AIM |
| *rs382259* | T/C | 6 | *Intergenic* | Intergenic | 16 | AIM |
| *rs12203592* | C/T | 6 | *IRF4* | Intron 4 | 16 | AIM |
| *rs354690* | T/C | 2 | *Intergenic* | Intergenic | 16 | AIM |
| *rs4988235* ^b^ | C/T | 2 | *MCM6* | Intron 13 | 17 | AIM |
| *rs12913832* | A/G | 15 | *HERC2* | Intron 87 | 18 | AIM |

^a^ Major allele/minor allele

^b^ This SNP was genotyped in 81.5 % of the samples and it was largely redundant with *rs6730157* (R^2^ = 0.84)

**References for Table S1:**

1. Nath SK, Han S, Kim-Howard X, Kelly JA, Viswanathan P, Gilkeson GS, Chen W, Zhu C, McEver RP, Kimberly RP, Alarcón-Riquelme ME, Vyse TJ, Li QZ, Wakeland EK, Merrill JT, James JA, Kaufman KM, Guthridge JM, Harley JB: A nonsynonymous functional variante in integrin-alpha(M) (encoded by ITGAM) is associated with systemic lupus erythematosus. *Nat Genet* 2008, 40:152-154.
2. International Consortium for Systemic Lupus Erythematosus Genetics (SLEGEN), Harley JB, Alarcón-Riquelme ME, Criswell LA, Jacob CO, Kimberly RP, Moser KL, Tsao BP, Vyse TJ, Langefeld CD, Nath SK, Guthridge JM, Cobb BL, Mirel DB, Marion MC, Williams AH, Divers J, Wang W, Frank SG, Namjou B, Gabriel SB, Lee AT, Gregersen PK, Behrens TW, Taylor KE, Fernando M, Zidovetzki R, Gaffney PM, Edberg JC, Rioux JD, et al.: *Genomewide association scan in women with systemic lupus erythematosus identifies susceptibility variants in ITGAM, PXK, KIAA1542 and other loci.* Nat Genet 2008, 40:204-210.
3. Hom G, Graham RR, Modrek B, Taylor KE, Ortmann W, Garnier S, Lee AT, Chung SA, Ferreira RC, Pant PV, Ballinger DG, Kosoy R, Demirci FY, Kamboh MI, Kao AH, Tian C, Gunnarsson I, Bengtsson AA, Rantapää-Dahlqvist S, Petri M, Manzi S, Seldin MF, Rönnblom L, Syvänen AC, Criswell LA, Gregersen PK, Behrens TW: *Association of systemic lupus erythematosus with C8orf13-BLK and ITGAM-ITGAX.* N Engl J Med 2008, 358:900-909.
4. Remmers EF, Plenge RM, Lee AT, Graham RR, Hom G, Behrens TW, de Bakker PI, Le JM, Lee HS, Batliwalla F, Li W, Masters SL, Booty MG, Carulli JP, Padyukov L, Alfredsson L, Klareskog L, Chen WV, Amos CI, Criswell LA, Seldin MF, Kastner DL, Gregersen PK: STAT4 and the risk of rheumatoid arthritis and systemic lupus erythematosus. *N Engl J Med* 2007, 357:977-986.
5. Kobayashi S, Ikari K, Kaneko H, Kochi Y, Yamamoto K, Shimane K, Nakamura Y, Toyama Y, Mochizuki T, Tsukahara S, Kawaguchi Y, Terai C, Hara M, Tomatsu T, Yamanaka H, Horiuchi T, Tao K, Yasutomo K, Hamada D, Yasui N, Inoue H, Itakura M, Okamoto H, Kamatani N, Momohara S: Association of STAT4 with susceptibility to rheumatoid arthritis and systemic lupus erythematosus in the Japanese population. *Arthritis Rheum* 2008, 58:1940-1946.
6. Palomino-Morales RJ, Rojas-Villarraga A, González CI, Ramírez G, Anaya JM, Martín J: STAT4 but not TRAF1/C5 variants influence the risk of developing rheumatoid arthritis and systemic lupus erythematosus in Colombians. *Genes Immun* 2008, 9:379-382.
7. Taylor KE, Remmers EF, Lee AT, Ortmann WA, Plenge RM, Tian C, Chung SA, Nititham J, Hom G, Kao AH, Demirci FY, Kamboh MI, Petri M, Manzi S, Kastner DL, Seldin MF, Gregersen PK, Behrens TW, Criswell LA: Specificity of the STAT4 genetic association for severe disease manifestations of systemic lupus erythematosus. *PLoS Genet* 2008, 4:e1000084.
8. Sigurdsson S, Nordmark G, Göring HH, Lindroos K, Wiman AC, Sturfelt G, Jönsen A, Rantapää-Dahlqvist S, Möller B, Kere J, Koskenmies S, Widén E, Eloranta ML, Julkunen H, Kristjansdottir H, Steinsson K, Alm G, Rönnblom L, Syvänen AC: Polymorphisms in the tyrosine kinase 2 and interferon regulatory factor 5 genes are associated with systemic lupus erythematosus. *Am J Hum Genet* 2005, 76:528-537.
9. Cunninghame Graham DS, Akil M, Vyse TJ: Association of polymorphisms

across the tyrosine kinase gene, TYK2 in UK SLE families. *Rheumatology (Oxford)* 2007, 46:927-930.

1. Sawalha AH, Webb R, Han S, Kelly JA, Kaufman KM, Kimberly RP, Alarcón-Riquelme ME, James JA, Vyse TJ, Gilkeson GS, Choi CB, Scofield RH, Bae SC, Nath SK, Harley JB: Common variants within MECP2 confer risk of systemic lupus erythematosus. *PLoS ONE* 2008, 3:e1727.
2. Kozyrev SV, Abelson AK, Wojcik J, Zaghlool A, Linga Reddy MV, Sanchez E, Gunnarsson I, Svenungsson E, Sturfelt G, Jönsen A, Truedsson L, Pons-Estel BA, Witte T, D'Alfonso S, Barizzone N, Danieli MG, Gutierrez C, Suarez A, Junker P, Laustrup H, González-Escribano MF, Martin J, Abderrahim H, Alarcón-Riquelme ME: Functional variants in the B-cell gene BANK1 are associated with systemic lupus erythematosus. *Nat Genet* 2008, 40:211-216.
3. Cunninghame Graham DS, Vyse TJ, Fortin PR, Montpetit A, Cai YC, Lim S, McKenzie T, Farwell L, Rhodes B, Chad L, Hudson TJ, Sharpe A, Terhorst C, Greenwood CM, Wither J, Rioux JD, CaNIOS GenES Investigators: Association of LY9 in UK and Canadian SLE families. *Genes Immun* 2008, 9:93-102.
4. Musone SL, Taylor KE, Lu TT, Nititham J, Ferreira RC, Ortmann W, Shifrin N, Petri MA, Kamboh MI, Manzi S, Seldin MF, Gregersen PK, Behrens TW, Ma A, Kwok PY, Criswell LA. *Multiple polymorphisms in the TNFAIP3 region are independently associated with systemic lupus erythematosus.* Nat Genet. 2008, 40(9):1062-4.
5. Gateva V, Sandling JK, Hom G, Taylor KE, Chung SA, Sun X, Ortmann W, Kosoy R, Ferreira RC, Nordmark G, Gunnarsson I, Svenungsson E, Padyukov L, Sturfelt G, Jönsen A, Bengtsson AA, Rantapää-Dahlqvist S, Baechler EC, Brown EE, Alarcón GS, Edberg JC, Ramsey-Goldman R, McGwin G Jr, Reveille JD, Vilá LM, Kimberly RP, Manzi S, Petri MA, Lee A, Gregersen PK, Seldin MF, Rönnblom L, Criswell LA, Syvänen AC, Behrens TW, Graham RR. *A large-scale replication study identifies TNIP1, PRDM1, JAZF1, UHRF1BP1 and IL10 as risk loci for systemic lupus erythematosus.* Nat Genet. 2009, 41:1228-33
6. Cunninghame Graham DS, Graham RR, Manku H, Wong AK, Whittaker JC, Gaffney PM, Moser KL, Rioux JD, Altshuler D, Behrens TW, Vyse TJ. *Polymorphism at the TNF superfamily gene TNFSF4 confers susceptibility to systemic lupus erythematosus.* Nat Genet. 2008, 40:83-9.
7. Tian C, Plenge RM, Ransom M, Lee A, Villoslada P, et al. (2008) *Analysis and application of European genetic substructure using 300 K SNP information.* PLoS Genet 4: e4. doi:10.1371/journal.pgen.0040004.
8. Han J, Kraft P, Nan H, Guo Q, Chen C, et al. (2008) *A Genome-Wide Association Study Identifies Novel Alleles Associated with Hair Color and Skin Pigmentation.* PLoS Genet 4: e1000074. doi:10.1371/journal.pgen.1000074.
9. Bersagleri T, Sabeti PC, Patterson N et al. *Genetic signatures of strong recent positive selection at the lactase gene.* Am J Hum Genet. 2004, 74:1111-20

**Figure S1:** Bias to a stronger association in Southern Europeans than in Central Europeans of 9 SLE loci from Suarez-Gestal et al. [24] Random effect meta-analysis O.R. for the risk allele and their 95 % C.I. are represented in descending order from left to right. *MECP2* data is only from women because this locus is in the X chromosome


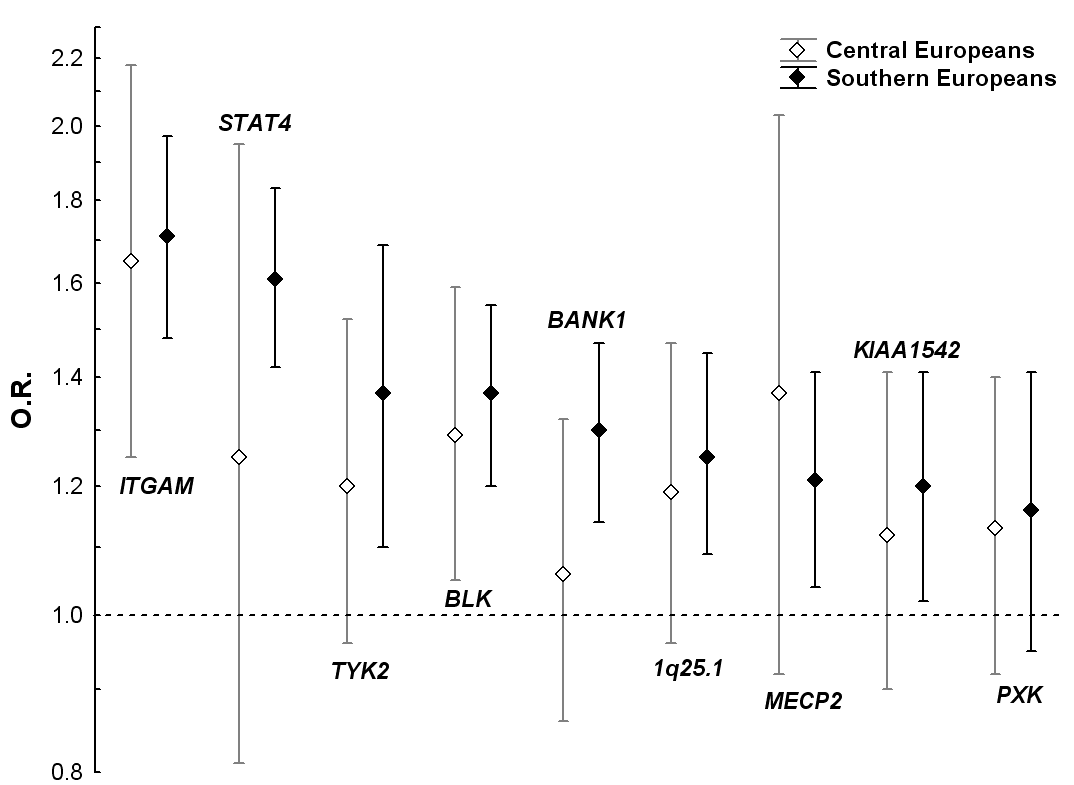


**Figure S2:** Comparison of the random effect meta-analysis O.R. for the 12 SNPs associated with SLE in the second phase of our study between the Central Europeans and Southern Europeans. O.R. for the risk allele and their 95 % C.I. are represented in descending order from left to right.


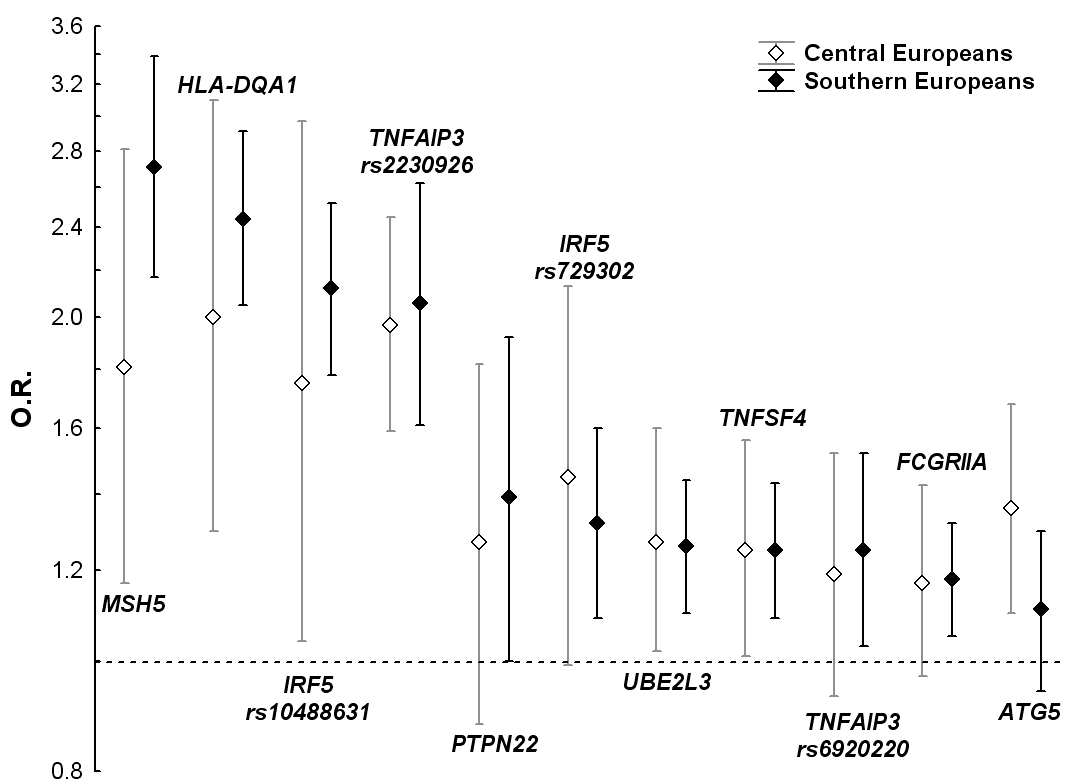

Supplement: Additional file 1 — Table S1 presenting a detailed description of all the SNPs included in the study, and Figures S1 and S2 with random effect meta-analysis results corresponding to the same data as Figure 1in the manuscript. [file ar3818-S1.DOC]
